# A New Cloud-Native Tool for Pharmacogenetic Analysis

**DOI:** 10.3390/genes15030352

**Published:** 2024-03-11

**Authors:** David Yu Yuan, Jun Hyuk Park, Zhenyu Li, Rohan Thomas, David M. Hwang, Lei Fu

**Affiliations:** 1 European Nucleotide Archive, European Bioinformatics Institute, European Molecular Biology Laboratory, Hinxton, Cambridge CB10 1SD, UK; 2 Department of Bioinformatics and Computational Biology, Faculty of Arts and Science, University of Toronto, Toronto, ON M5S 3G3, Canada; 3 Department of Laboratory Medicine & Pathobiology, Faculty of Medicine, University of Toronto, Toronto, ON M5S 1A8, Canada; 4 Precision Diagnostics and Therapeutics Program, Sunnybrook Health Sciences Centre, Toronto, ON M4N 3M5, Canada; 5 Sunnybrook Research Institute, Toronto, ON M4N 3M5, Canada

**Keywords:** pharmacogenetics, bioinformatics pipeline, cloud-native technologies, workflow, genomic data analysis

## Abstract

Background: The advancement of next-generation sequencing (NGS) technologies provides opportunities for large-scale Pharmacogenetic (PGx) studies and pre-emptive PGx testing to cover a wide range of genotypes present in diverse populations. However, NGS-based PGx testing is limited by the lack of comprehensive computational tools to support genetic data analysis and clinical decisions. Methods: Bioinformatics utilities specialized for human genomics and the latest cloud-based technologies were used to develop a bioinformatics pipeline for analyzing the genomic sequence data and reporting PGx genotypes. A database was created and integrated in the pipeline for filtering the actionable PGx variants and clinical interpretations. Strict quality verification procedures were conducted on variant calls with the whole genome sequencing (WGS) dataset of the 1000 Genomes Project (G1K). The accuracy of PGx allele identification was validated using the WGS dataset of the Pharmacogenetics Reference Materials from the Centers for Disease Control and Prevention (CDC). Results: The newly created bioinformatics pipeline, Pgxtools, can analyze genomic sequence data, identify actionable variants in 13 PGx relevant genes, and generate reports annotated with specific interpretations and recommendations based on clinical practice guidelines. Verified with two independent methods, we have found that Pgxtools consistently identifies variants more accurately than the results in the G1K dataset on GRCh37 and GRCh38. Conclusions: Pgxtools provides an integrated workflow for large-scale genomic data analysis and PGx clinical decision support. Implemented with cloud-native technologies, it is highly portable in a wide variety of environments from a single laptop to High-Performance Computing (HPC) clusters and cloud platforms for different production scales and requirements.

## 1. Introduction

Individuals vary significantly in their drug responses and treatment outcomes. The same dosage, effective in some patients, may inevitably be ineffective and may even cause adverse drug reactions (ADRs) in others. Studies have indicated that ADRs have been an important cause of hospital admissions and in-hospital mortality [1,2]. Many factors can contribute to this interindividual variability, such as genetic variations in drug metabolizing enzymes and transporters, drug–drug interactions, diet–drug interactions, physiological conditions, concurrent diseases, as well as environmental exposures [3]. Pharmacogenetic (PGx) tests translate germ-line genotypes into actionable phenotypes and provide recommendations on dosing of medications. Aiming to optimize drug therapy, prevent ADRs, and improve patient safety, some PGx tests have been successfully implemented clinically in a single-gene-drug-pair approach [4,5,6]. This reactive testing approach has limited capacity, fixed coverage, bias in variant selection, and may delay treatment while waiting for the PGx test result. The US FDA Table of Pharmacogenetic Associations lists more than 100 gene-drug pairs and their interactions [7]. The Clinical Pharmacogenetics Implementation Consortium (CPIC), a shared project between the Pharmacogenomics Knowledge Base (PharmGKB) and the National Institute of Health (NIH) Pharmacogenomics Research Network (PGRN), and the Dutch Pharmacogenetics Working Group (DPWG) have developed guidelines on genotype-guided drug therapy [8]. The CPIC database contains 517 gene-drug pairs in v 1.36.0 as of December 2023. To meet the increasing clinical demand, pre-emptive testing would be an ideal strategy to generate variant data for multiple genes before prescribing any target drugs [9,10]. Recently, a large-scale multicenter implementation study, PREPARE, has demonstrated that genotype-guided drug prescription using a 12-gene PGx panel can reduce the incidence of clinically relevant ADRs significantly and improve the safety of drug therapy [11].

With the advancement of next-generation sequencing (NGS) technologies, the volume of genomic data has increased dramatically. The availability of genomic data provides opportunities for large-scale PGx studies and pre-emptive PGx testing. Bioinformatics pipelines are needed to analyze the genomic sequencing data to report the actionable PGx variants efficiently and reliably, and to integrate the PGx results into electronic health records to deliver the clinical decision support at the point of prescribing [12,13,14]. Thus, bioinformatics pipelines become essential tools to use genomic data to its full potential and to interpret it for clinical applications [15,16]. In this study, we aimed to develop a cloud-based bioinformatics pipeline for PGx testing covering the entire workflow including NGS data analysis, variant allele assignment, genotype interpretation, and clinical decision support.

## 2. Methods

### 2.1. Creating a PGx Database

The purpose of creating a PGx database was to provide a filter system for the pipeline to narrow the sequence analysis to PGx relevant gene variants as well as corresponding interpretations for these variants, such as clinical significance and dosing recommendations. The database contained the following attributes: gene name, drug name, genotype alias, variant position relative to the latest human reference genomes GRCh37 and GRCh38, variant cDNA, amino acid change, enzyme activity or variant effect, rs number (a.k.a. reference single nucleotide polymorphism (SNP) ID), genotype code, variants result, interpretation, and dosing recommendations. Each row of the PGx database represented a diplotype. Information regarding clinical significance was drawn mainly from the CPIC guidelines [17].

### 2.2. Designing a Bioinformatics Pipeline

**Figure 1:**
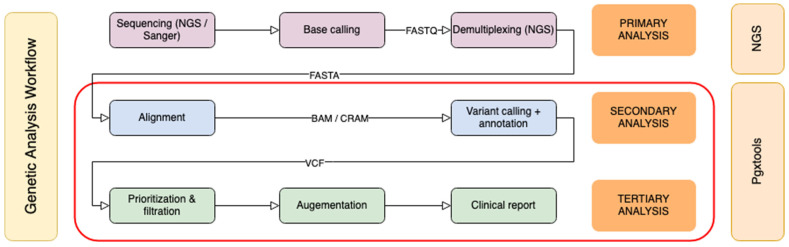
Genomic sequence analysis workflow. The secondary and tertiary analysis steps in the red box are covered by Pgxtools.

Genomic sequence analysis can be divided into three phases: primary analysis, secondary analysis, and tertiary analysis (Figure 1). The primary analysis is usually completed on the sequencer. Classical bioinformatics pipelines focus on secondary analysis to create sequence alignments and to identify variants. Our pipeline was designed to cover both secondary and tertiary analysis. The starting point was either a Binary Alignment Map (BAM)/Compressed Reference-oriented Alignment Map (CRAM) or Variant Calling Format (VCF) input files from targeted sequencing panels or from WGS.

With our pipeline, named Pgxtools, 20–100 thousand variants per exome or 3–4 million variants per genome can be prioritized and filtered to be comprehensible by healthcare providers. Variants with known clinical significance were annotated with suggestions and references to PGx guidelines. The final clinical report contained tertiary analysis results specific to PGx testing. Our PGx database and pipeline formed an automated workflow to provide PGx recommendations specific to patient genetic profiles.

### 2.3. Validating Variant Calls and Variant Allele Assignment

To verify the quality of variant calls by Pgxtools, we used Pgxtools to analyze the WGS alignments (BAM/CRAM) in the 1000 Genomes Project (G1K) dataset (https://www.internationalgenome.org/1000-genomes-summary/, accessed on 15 January 2023), and compared the variants reported by Pgxtools with the variants documented in the G1K dataset. To further investigate the discordance in variant calls between our pipeline and the G1K datasets, we employed two independent methods, Samtools view command and EnsEMBL genome browser, to identify which results were correct.

To validate the accuracy of variant to genotype mapping, we used PGx reference materials with “Consensus genotypes for 28 PGx genes” [18], archived on CDC [19] as a gold standard. We used Pgxtools to analyze all 70 WGS alignments used in the PGx reference materials archived in the European Nucleotide Archive (ENA) [20] and reported the genotypes of the PGx genes. The genotype results called by Pgxtools were compared with the consensus genotypes published on this CDC dataset.

## 3. Results

### 3.1. System Architecture

Our newly developed pipeline, Pgxtools, is designed with the latest technologies to be completely cloud-native and highly portable. The pipeline is containerized so it can run on Kubernetes clusters in different clouds without changes. We have used Kubernetes on MacBook Pro for development, and Google Cloud Platform (GCP) and High-Performance Computing (HPC) clusters at Genomics England for production.

**Figure 2:**
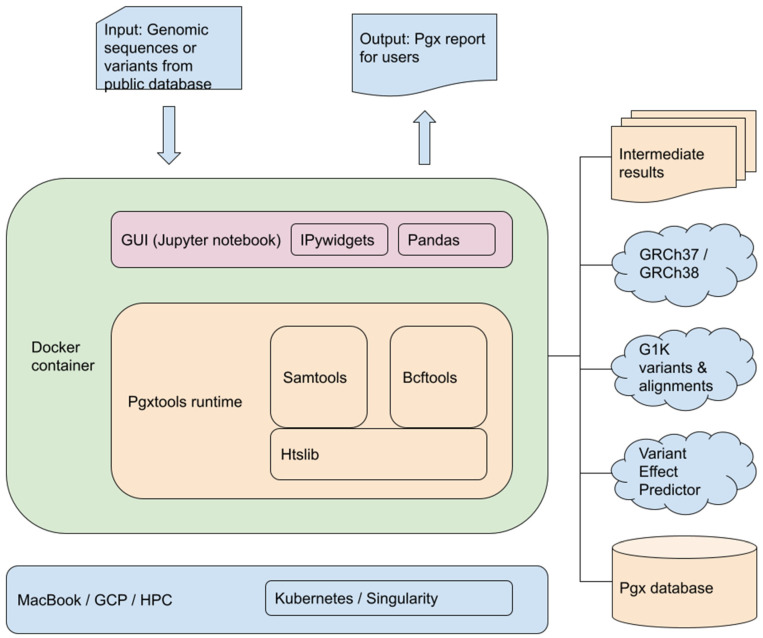
Components and architecture of Pgxtools.

Pgxtools has a front-end which consists of a Graphical User Interface (GUI) on a Jupyter notebook server augmented with IPywidgets and Pandas. It has a back-end runtime with Samtools and Bcftools based on Htslib. The Docker container is stateless by design. The data are either stored on a persistent volume managed by Kubernetes or from the various sources in the cloud or on storage volumes in HPC as described below (Figure 2).

The input of genomic alignment maps or variants, the output of PGx reports, and the intermediate results are stored on a persistent volume outside of the container. They can survive the events of the container upgrade, shutdown, eviction, etc. The two human reference genomes, GRCh37 and the latest GRCh38 are downloaded from a public File Transfer Protocol (FTP) site at the European Bioinformatics Institute, European Molecular Biology Laboratory (EMBL-EBI). The G1K variants and alignments for GRCh37 and GRCh38 are accessed directly from an S3 bucket for public data on Amazon Web Services (AWS). The public Abstract Programming Interface (API) of the Variant Effect Predictor (VEP) at EMBL-EBI is used to obtain the details of PGx variants when the Docker image of the pipeline is built. Datashim is used to bridge the cloud object store and Linux POSIX filesystem.

We created a database including 13 genes and the most common drugs with ADRs caused by the variants in these genes (Table 1). The genes and drugs were selected based on information in CPIC guidelines. The genes include *COMT*, *CYP2B6*, *CYP2C9*, *CYP2C19*, *CYP2D6*, *CYP3A5*, *CYP4F2*, *DPYD*, *IL28B*, *NUDT15*, *SLCO1B1*, *TPMT*, and *VKORC1*. Detailed information of the variants can be found in Supplemental Table S1. This database is stored in the Docker image and used by the pipeline to perform targeted analysis. The Pgxtools references the database of the genes and drugs with the variant-specific interpretation and compares them against the genes and variants identified from the input sequence data. The tool generates specific PGx recommendations for the given genetic information.

### 3.2. Accuracy of Variant Calls from Alignments to Variants

We used the variants in the G1K dataset as the gold standard to verify the quality of the variants called by Pgxtools from the sequence alignments (BAM/CRAM). We ran Pgxtools on 2504 WGS sample alignments on GRCh37 and 3200 WGS sample alignments on GRCh38 in the G1K dataset. There were at least 92.69% of samples with variants reported correctly on GRCh37 among all 13 genes in our study except for *CYP2D6* (see the GRCh37 row in Table 2). On GRCh38, Pgxtools reported 100% of variants matching the results in the G1K dataset except for *CYP2D6*. For *CYP2D6*, the concordance improved from 68.17% on GRCh37 to 98.59% on GRCh38 (Table 2).

We suspected that the discordance in variants reported by Pgxtools and the G1K project among the genes of *CYP2D6*, *NUDT15*, *DPYD*, *TPMT*, and *CYP3A5* were most likely caused by the quality of BAM files on GRCh37 in the G1K dataset. To test this hypothesis, we created synthetic data by backporting the VCF and CRAM files with these genes from GRCh38 in the G1K dataset to GRCh37 coordinated with CrossMap [21]. We ran Pgxtools on the backported CRAM files to compare the variants of the synthetic alignments. The concordance improved to 97.84–100% from 68.17 to 98.80% (see the row of “GRCh38 to GRch37 backported” in Table 2), proving that Pgxtools identifies variants correctly with both reference genomes of GRCh37 and GRCh38. The sample alignments (BAMs/CRAMs) for these genes on GRCh37 were the major limiting factor causing the discordance in variants reported.

We conducted detailed analysis on discordance in variant calling by Pgxtools and the G1K dataset. We employed two independent methods, Samtools view command and EnsEMBL genome browser, to identify which variant calling results were correct.

**Figure 3:**
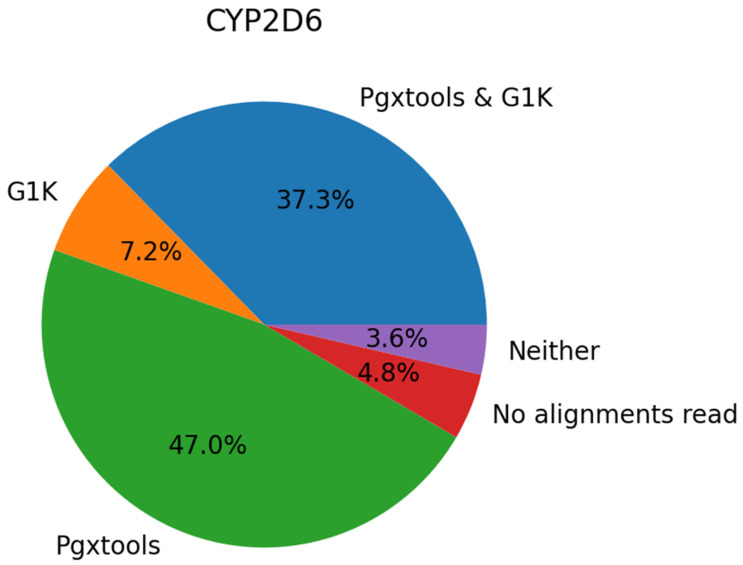
Comparison of Pgxtools and G1K results in 45 samples with 83 variants in the *CYP2D6* gene on GRCh38. Percentages of variants correctly called by Pgxtools alone (green), G1K alone (orange), both Pgxtools and G1K (blue), or neither Pgxtools nor G1K (purple) are depicted. Variants without contigs for variant calling are depicted in red. (Due to the rounding error, the total is 99.9%.).

On GRCh38, there were a total of 45 samples with 83 variant calls in *CYP2D6* showing significant discordances between Pgxtools and G1K. With visual inspection of the 45 samples in EnsEMBL at EMBL-EBI, we concluded that 39 of the 83 variants (47.0%) were called correctly by Pgxtools alone, 6 variants (7.2%) were called correctly by G1K alone, and 31 variants (37.3%) were called correctly by both Pgxtools and G1K. There were also four variants (4.8%) without contigs for variant calling and three variants (3.6%) were called incorrectly by both Pgxtools and G1K (Figure 3). Overall, Pgxtools made the correct variant calls most of the time, performing significantly better than the variant calling in the G1K dataset (84.3% vs. 44.5%, respectively).

As a side note, the *CYP2D6* gene is known to be difficult for sequencing with NGS. The coverage can vary significantly from contig to contig, and some contigs contain very ambiguous base calls (e.g., the G1K sample alignments NA19210.final.cram around 42,130,692 as shown in Supplemental Figure S1), making variant calling difficult and less accurate. Our data showed that Pgxtools produced much better results for *CYP2D6* gene variant calling.

**Figure 4:**
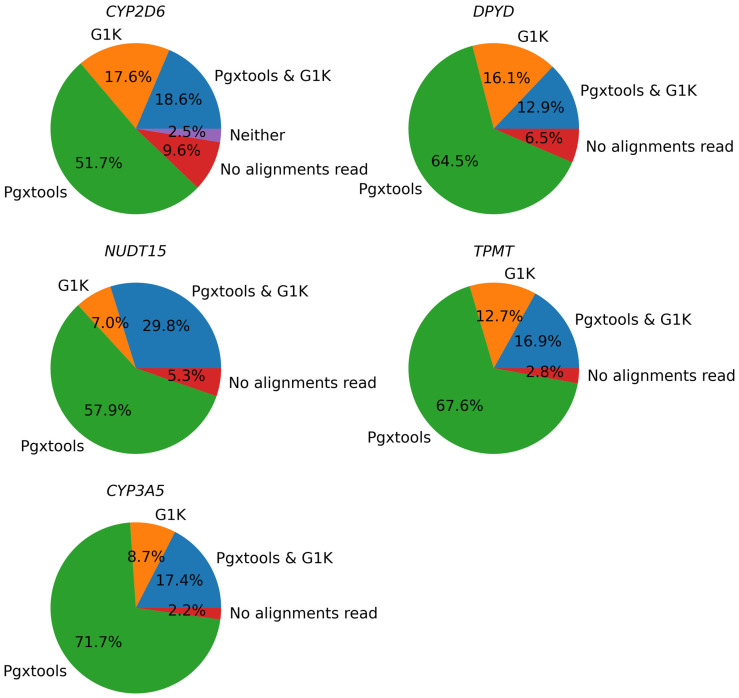
Comparison of Pgxtools and G1K results in WGS samples with 1484 variants within the *CYP2D6*, *NUDT15*, *DPYD*, *TPMT*, and *CYP3A5* genes on GRCh37.

There were 1132 samples with 1484 variants showing significant discordances between G1K and Pgxtools results on *CYP2D6*, *NUDT15*, *DPYD*, *TPMT*, and *CYP3A5* on GRCh37. We conducted a manual inspection on sample alignment files with the Samtools view command. We concluded that Pgxtools was reporting variant existence with 70.3%, 87.7%, 77.4%, 84.5%, and 89.1% accuracy in *CYP2D6*, *NUDT15*, *DPYD*, *TPMT*, and *CYP3A5*, respectively. In contrast, G1K was reporting variants with 36.2%, 36.8%, 29.0%, 29.6%, and 26.1% accuracy in *CYP2D6*, *NUDT15*, *DPYD*, *TPMT*, and *CYP3A5*, respectively (Figure 4). However, there were significant numbers of sample files without alignments in variant positions on GRCh37, especially in *CYP2D6* (9.6%), which led to reduced accuracy of variant findings.

As shown in the detailed analysis above, Pgxtools demonstrated much higher accuracy in variant calling for these genes. The G1K dataset on GRCh38 can be used as the gold standard but the dataset on GRCh37 is suboptimal for some genes.

### 3.3. From Variants to Genotypes

**Figure 5:**
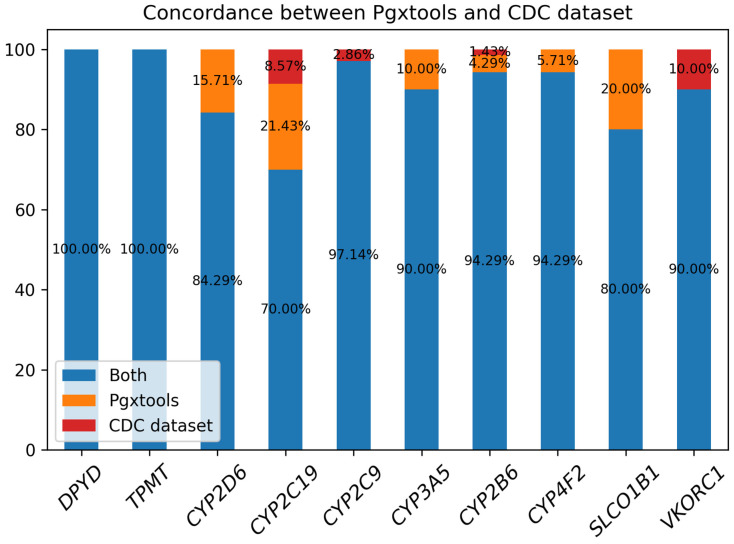
The concordance between Pgxtools and the community consensus genotypes for the selected PGx genes with the CDC PGx reference materials.

We used a consensus-based community standard, “Consensus genotypes for 28 PGx genes” [18], archived on CDC [19], to verify the variant to genotype mapping by Pgxtools. There were three genes not included in the CDC 28 PGx gene panel: *COMT*, *NUDT15*, and *IL28B*. We analyzed the concordance of the genotype calling by Pgxtools and the CDC dataset on the remaining 10 genes. We used WGS BAM alignments from all 70 PGx reference materials by the original study archived in ENA [20]. The results showed remarkable concordance rates between Pgxtools and the community consensus (Figure 5).

There was 100% concordance of genotype calling for the genes of *DPYD* and *TPMT* between Pgxtools and the CDC dataset. For the other 8 genes, Pgxtools reported genotypes 100% correctly in the *CYP2D6*, *CYP3A5*, *CYP4F2*, and *SLCO1B1* genes according to the available WGS alignment. However, there were 15.71%, 10.00%, 5.71%, and 20.00% discordant genotype calls in these genes compared with the consensus genotype assignment. For *CYP2C19* and *CYP2B6*, Pgxtools reported genotypes 91.43% and 98.58% correctly after manual verification with WGS alignment; however, the remaining 8.57% and 1.42% of the genotypes were assigned incorrectly by Pgxtools after manual verification. The reason is still under investigation. For *CYP2C9* and *VKORC1*, Pgxtools was 97.14% and 90.00% correct whereas the community consensus was 100% correct, indicating the need to further optimize the Pgxtools analysis on these two genes to improve concordance.

### 3.4. PGx Interpretation and Reports

Pgxtools analyzes WGS or targeted sequences the same way via the same user interfaces, command line interface (CLI), or graphic user interface (GUI) with alignments or variant calls as input. It can generate reports including gene-drug pairs, their corresponding PGx phenotype prediction, and clinical decision support, such as dosing recommendations. On the user interface, the operator can choose gene(s) from their drop-down lists and then create a customized report (Supplemental Figure S2). The accuracy of the tertiary analysis and reporting has been manually verified and cross-checked for consistency with the clinical practice guidelines used for building our database.

In a report for a particular single-gene-drug-pair format (Supplemental Figure S3), Pgxtools provides detailed analysis to support the PGx decisions. It lists all variants with clinical significance in that gene. For each variant, it reports nucleotide change, amino acid change, and genotype. It also reports the actionable PGx interpretation of such changes. The comments of the gene variants and reference documentation are also included for further investigation.

Pgxtools places more emphasis on large-scale pre-emptive PGx testing. To provide a complete overview of potential PGx implications, Pgxtools can generate a report format with a high-level summary of all genes and variants identified from the input sequencing data. This format does not include PGx interpretation or dosing recommendations (Supplemental Figure S4). If detailed PGx decision support is needed, the most comprehensive all-gene-all-drug report format can be selected from the user interface. This report organizes the details by gene. For each gene, all the relevant variants are listed with the same level of detail as described in the above single-gene-drug-pair report format.

## 4. Discussion

In this article, we report on the creation of a PGx database and a pipeline to perform both secondary and tertiary analysis of genomic sequences from targeted sequencing panels or WGS on human reference genomes GRCh37 and GRCh38. The database and pipeline are fully integrated to form a PGx clinical decision support workflow, which is implemented with cloud-native technologies to be highly portable in a wide variety of environments from a single laptop, to GCP, to HPC clusters for different production scales and requirements.

The pipeline, Pgxtools, takes NGS data such as BAM or VCF files as the input. It then generates the PGx reports specific to the patient’s genetic or genomic profile filtered by the genes and drugs specified by the operator of the tool. Pgxtools provides a list of genes for the operator to select one or all genes in the database. The tool then displays a list of drugs based on the gene or genes selected by the operator. The reports generated by Pgxtools are only relevant to the gene(s) and drug(s) selected by the operator. A Pgxtools report contains the identified gene and allele variants as well as the specific interpretations and recommendations based on clinical practice guidelines.

We conducted strict quality verification analysis against our pipeline with 5704 human genome sequences on both GRCh37 and GRCh38. We demonstrated that the Pgxtools secondary analyses are highly reliable and accurate. In addition, we confirmed that the G1K dataset on GRCh38 can be used as the gold standard for PGx studies, while the G1K dataset on GRCh37 has lower accuracy and is not suitable for this purpose. With two independent methods, we demonstrated that Pgxtools reports more accurate results in the 13 PGx genes than the G1K dataset on GRCh37 and GRCh38.

During this investigation, we encountered a number of challenges. First, each CPIC guideline was updated periodically on the CPIC website, and the latest publication may not reflect the most updated changes. Our database was created based on the original published guidelines. To follow the most updated therapeutic recommendations and allele definitions, we checked the CPIC and Pharmvar websites periodically and amended our database accordingly.

Second, the CPIC guidelines determined therapeutic recommendations based on evidence from functional and clinical data and/or other existing guidelines. Based on the strength of evidence, CPIC guidelines assigned “strong”, “moderate”, “optional”, or “no recommendation” to their recommendations [22]. To indicate the strengths of evidence, we kept the CPIC classification system in the PGx database and indicated this in brackets in front of each interpretation in the PGx database and in the report.

Third, we found some inconsistencies translating genotype to phenotype among guidelines for different target drugs. For example, for *CYP2C19*, we observed that in the CPIC guideline for selective serotonin reuptake inhibitors published in 2015, both the *17/*17 and *1/*17 genotypes were defined as ultrarapid metabolizers [23]. However, in the newer guidelines for clopidogrel, proton pump inhibitors, tricyclic antidepressants, and voriconazole, *1/*17 was defined as a rapid metabolizer [23,24,25,26]. According to DPWG, *1/*17 was defined as a normal metabolizer because of a relatively small increase in the enzyme activity effect of the *17 allele [27]. We defined *1/*17 as a rapid metabolizer in our PGx database because the *17 and *1 alleles did have statistical differences in terms of pharmacokinetic parameters [26]. Furthermore, since therapeutic recommendations for normal and rapid metabolizers of *CYP2C19* in CPIC guidelines were the same, the phenotypic definition of *1/*17 did not affect its drug recommendation.

For *CYP2D6*, we observed that in the guidelines for selective serotonin reuptake inhibitors, tamoxifen, atomoxetine, tricyclic antidepressants, and ondansetron/tropisetron, the allele *10 was given an activity score of 0.5 and the genotype *10/*10 was defined as a normal metabolizer [23,28,29,30,31]. However, in the guideline for opioid, *10 was given an activity score of 0.25 and *10/*10 was defined as an intermediate metabolizer [32]. Due to inconsistencies in translating *CYP2D6* genotype to phenotype across different laboratories and guidelines, CPIC used a modified Delphi method to obtain a consensus for translating the *CYP2D6* genotype to phenotype among a panel of international experts [33]. As a result of the consensus, CPIC modified the activity score of *10 from 0.5 to 0.25 and changed the definition of metabolizers based on the activity score as follows: ultrarapid metabolizer was changed from over 2 to over 2.25; normal metabolizer was changed from between 1 and 2 to between 1.25 and 2.25; and intermediate metabolizer was changed from 0.5 to between 0 and 1.25 [33]. Thus, the phenotype assignment for *10/*10 was an intermediate metabolizer as the genotype had an activity score of 0.5 [33]. We adjusted interpretations in the PGx database based on the latest consensus on the translation of *CYP2D6* genotype to phenotype.

Furthermore, *CYP2D6* is a polymorphic gene with over 100 known allelic variants [29]. We noticed that the CPIC guidelines did not categorize *CYP2D6* alleles based on their frequency, clinical relevance, or the amount of evidence. On the other hand, the Association for Molecular Pathology (AMP) guideline provided a “two tier” system for the allelic variants based on a set of criteria [34]. Our PGx database included all the variants covered in the “two tier system” described in the AMP guideline to reflect the importance of these variants.

The majority of clinically implemented PGx tests and commercially available PGx panels are targeted genotype methods covering a limited number of variants in each gene. The coverage of alleles may not be sufficient in diverse populations. In addition, low-frequency no-function alleles not included in the testing panel will give false classification if the patient happens to be a carrier of that variant. The NGS-based PGx test provides opportunities to fill these gaps by analyzing all PGx relevant variants in the gene. The G1K dataset describes common human genetic variants in a diverse set of thousands of individuals from 26 populations [35]. Our new pipeline, Pgxtools, analyzes a total of 5704 individual WGS (2504 on GRCh37 and 3200 on GRCh38) in the G1K dataset efficiently and accurately. For future development, we plan to expand the capacity of our pipeline to be able to report all actionable variants currently well-characterized in the CPIC database regardless of their allele frequencies and population background.

Currently, our pipeline is limited to analyzing the variants with rs ID in the SNP database. We plan to develop another branch of tools to analyze gene structural changes and variants without rs ID, such as *CYP2D6* gene duplication/deletion and PGx-associated HLA typing. In addition, the current version of Pgxtools only allows a selection of gene(s) and then drug(s) to initiate analysis and reporting. We are considering developing additional features to allow operators to use drug name(s) and then gene(s) to order analysis. Drug–drug interaction is an important aspect affecting drug responses and clinical outcomes. In future development, this should be taken into consideration when multiple drugs are selected.

This paper describes the first iteration of Pgxtools. The main objective of the project was to create a tool for Pharmacogenetic analysis meeting our current research and clinical needs. In addition, we aim to make Pgxtools available to others in the future as an alternative to some existing PGx star allele calling pipelines, such as Aldy, PharmCAT, Stargazer, Cyrius, and StellarPGx [14,36,37,38,39]. A systematic review published by Twesigomwe, D. et al. (2020) provided a comprehensive comparison of multiple existing pipelines [40].

In summary, we have created a PGx database and pipeline for secondary and tertiary analysis of genomic sequence data that are fully integrated to form the basis of a PGx clinical decision support workflow. The workflow is implemented with cloud-native technologies to be highly portable. Our study reported here demonstrates that it is not only possible but also feasible to support the NGS-based large-scale pre-emptive PGx testing.

## Figures and Tables

**Table 1:** Genes and drugs for PGx analysis.

Gene	Drug(s)
*COMT*	Opioid
*CYP2B6*	Efavirenz
*CYP2C9*	Warfarin
*CYP2C19*	Clopidogrel, Proton Pump Inhibitors (Omeprazole, Lansoprazole, Pantoprazole, and Dexlansoprazole), Selective Serotonin Reuptake Inhibitors (Citalopram, Escitalopram, and Sertraline), Tricyclic Antidepressants (Tertiary Amines Amitriptyline, Clomipramine, Doxepin, Imipramine, and Trimipramine), Voriconazole
*CYP2D6*	Ondansetron and Tropisetron, Selective Serotonin Reuptake Inhibitors (Paroxetine and Fluvoxamine), Opioid (Codeine, Tramadol, and Hydrocodone), Atomoxetine, Tricyclic Antidepressants, Tamoxifen
*CYP3A5*	Tacrolimus
*CYP4F2*	Warfarin
*DPYD*	Fluoropyrimidines 5-fluorouracil
*IL28B*	PEG Interferon-α-Based Regimens
*NUDT15*	Thiopurine
*SLCO1B1*	Simvastatin
*TPMT*	Thiopurine (thioguanine, mercaptopurine, and azathioprine)
*VKORC1*	Warfarin

**Table 2:** Concordance of the variant calls by Pgxtools compared against the G1K dataset.

	*CYP2D6*	*CYP3A5*	*DPYD*	*NUDT15*	*TPMT*
GRCh37	68.17%	92.69%	98.80%	97.72%	97.40%
GRCh38	98.59%	100%	100%	100%	100%
GRCh38 to GRCh37 backported	97.84%	100%	99.84%	99.81%	99.56%

There was 100% concordance in the genes, *COMT*, *CYP2B6*, *CYP2C9*, *CYP2C19*, *CYP4F2*, *IL28B*, *SLCO1B1*, and *VKORC1* on both GRCh37 and GRCh38 in the 13 genes analyzed. Therefore, they are not listed in the table.

## Data Availability

Data are contained within the article and Supplementary Materials. In addition, the following public datasets are reused: The 1000 Genomes Project (G1K) dataset (https://www.internationalgenome.org/1000-genomes-summary/, accessed on 15 January 2023) The Reference Materials for Pharmacogenetics|CDC. Consensus Genotypes for 28 PGx Genes Archived on CDC as Part of the Reference Materials for Pharmacogenetics (https://www.cdc.gov/labquality/get-rm/inherited-genetic-diseases-pharmacogenetics/pharmacogenetics.html, accessed on 15 January 2023)

## Supplementary Materials

The following supporting information can be downloaded at: https://www.mdpi.com/article/10.3390/genes15030352/s1, Figure S1: G1K sample alignments NA19210.final.cram around 42130692; Figure S2: Graphic user interface of Pgxtools; Figure S3: Pharmacogenetics report on *TPMT* and thiopurine pair for sample HG01183; Figure S4: Pharmacogenetics summary report on all PGx gene variants found in sample HG01183; Table S1: Genes and variants for PGx analysis.
