# Supplementary material for: A New Cloud-Native Tool for Pharmacogenetic Analysis
Source: Genes (Basel). 2024 Mar 11;15(3):352. doi: 10.3390/genes15030352 (PMC10969787; doi:10.3390/genes15030352)
Supplement: Supplementary file 1 [file genes-15-00352-s001.zip › [Supplemental Figure S3] HG01183_basic_report_TPMT_Thiopurine Azathioprine.html.pdf]

MOLECULAR TEST REPORT

Sample ID:	HG01183	Source of Data:	HG01183.final.cram

Sample Type: BAM

Test Requested:

- Gene: TPMT
- Drug: Thiopurine Azathioprine

Method:
Method1

Variants Identified:

1. rs1142345-2&rs1800460-1

   Star allele:
   *3A/*3C

   Result(s):
   TPMT c.460G>A (p.Ala154Thr), HOMOZYGOUS MUTANT; TPMT c.719A>G (p.Tyr240Cys), HETEROZYGOUS

   Interpretation:
   (Strong) THE PATIENT'S TPMT GENOTYPE IS *3A/*3C. The *3A/*3C genotype, with variations involving both chromosomes, is often associated with low TPMT activity, extremely high concentrations of TGN metabolites, fatal toxicity without dose decrease, no MeTIMP metabolites and greatly increased risk of thiopurine-related leukopenia, neutropenia and myelosuppression. For nonmalignant conditions, consider alternative nonthiopurine immunosuppressant therapy. For malignancy, start with drastically reduced doses (reduce daily dose by 10-fold and dose thrice weekly instead of daily) and adjust doses of azathioprine based on the degree of myelosuppression and disease-specific guidelines. Allow 4–6 weeks to reach a steady-state after each dose adjustment.

Comments:
To date, more than 30 TPMT mutant alleles responsible for the enzyme deficiency have been described. The 3 common variations (c.238G>C, c.460G>A and c.719A>G) account for most Caucasians with low or intermediate TPMT activity. TPMT genotypes provide part of the information required to determine drug dosage. In special cases, a direct biochemical assay of the TPMT enzyme or its metabolites may be

indicated, and clinicians should monitor initial therapy carefully. TGN: thioguanine nucleotides. MeTIMP: metabolites of thiopurine methyltransferase. References: Schaeffeler E et al. Pharmacogenetics 2004;14:407-417 & Dervieux T et al. Clin Chem 2005;51:2074-2084. Relling MV et al. Clinical Pharmacogenetics Implementation Consortium Guideline for Thiopurine Dosing Based on TPMT and NUDT15 Genotypes: 2018 Update. Clin Pharmacol Ther. 2019 May;105(5):1095-1105. doi: 10.1002/cpt.1304.

Disclaimer:

This report assumes that the sample received is representative of the patient's genomic DNA.

The interpretation of this result may be dependent on genetic analysis, medical history and relevant family structure. This result should therefore be interpreted by appropriately qualified clinical personnel.

Consent should be obtained from the patient for release of these results.

Report Date and Time (GMT): 10/19/2022, 23:38

PGX Pipeline Version: 1.15.1

Operator Name: Test1
