# Supplementary material for: A New Cloud-Native Tool for Pharmacogenetic Analysis
Source: Genes (Basel). 2024 Mar 11;15(3):352. doi: 10.3390/genes15030352 (PMC10969787; doi:10.3390/genes15030352)
Supplement: Supplementary file 1 [file genes-15-00352-s001.zip › [Supplemental Figure S4] HG01183_genes_summary_report.html.pdf]

MOLECULAR TEST REPORT

Sample ID: HG01183 Source of Data: HG011831.final.cram

Genes summary report

Sample Type: BAM

Test Requested:

- Gene: COMT, CYP2B6, CYP2C19, CYP2C9, CYP2D6, CYP3A4, CYP3A5, CYP4F2, DPYD, IL28B, NUDT15, SLCO1B1, TPMT, VKORC1
- Drug: Atomoxetine, Clopidogrel, Efavirenz, Fluoropyrimidine 5-Fluorouracil, Fluoropyrimidine Capecitabine, Ondansetron , Opioid, Opioid Codeine, Opioid Hydrocodone, Opioid Tramadol, PEG Interferon-Alpha-Based Regimens, Proton Pump Inhibitor Dexlansoprazole, Proton Pump Inhibitor Lansoprazole, Proton Pump Inhibitor Omeprazole, Proton Pump Inhibitor Pantoprazole, Selective Serotonin Reuptake Inhibitor Citalopram, Selective Serotonin Reuptake Inhibitor Escitalopram, Selective Serotonin Reuptake Inhibitor Fluvoxamine, Selective Serotonin Reuptake Inhibitor Paroxetine, Selective Serotonin Reuptake Inhibitor Sertraline, Simvastatin, Tacrolimus, Tamoxifen, Thiopurine Azathioprine, Thiopurine Mercaptopurine, Thiopurine Thioguanine, Tricyclic Antidepressant Amitriptyline, Tricyclic Antidepressant Clomipramine, Tricyclic Antidepressant Doxepin, Tricyclic Antidepressant Imipramine, Tricyclic Antidepressant Trimipramine, Tricyclic Antidepressants, Tropisetron, Voriconazole, Warfarin

Method:
Method

Variants Identified:

1. COMT
   No variant detected
2. CYP2B6
   No variant detected
3. CYP2C19
   No variant detected
4. CYP2C9

   Final allele:
   *1/*3

   *1/*3, rs1057910-1, Warfarin, CYP2C9 c.1075A>C (p.Ile359Leu) HETEROZYGOUS
5. CYP2D6
   No variant detected

6. CYP3A4

   No variant detected
7. CYP3A5

   Final allele:

   *1/*3

   *1/*3, rs776746-1, Tacrolimus, CYP3A5 c.219-237A>G HETEROZYGOUS
8. CYP4F2

   No variant detected
9. DPYD

   No variant detected
10. IL28B

    No variant detected
11. NUDT15

    No variant detected
12. SLCO1B1

    No variant detected
13. TPMT

    Final allele:

    *3A/*3C

    *3A/*3C, rs1142345-2&rs1800460-1, Thiopurine Azathioprine, TPMT c.460G>A (p.Ala154Thr), HOMOZYGOUS MUTANT; TPMT c.719A>G (p.Tyr240Cys), HETEROZYGOUS
14. VKORC1

    No variant detected

Disclaimer:

This report assumes that the sample received is representative of the patient's genomic DNA.

The interpretation of this result may be dependent on genetic analysis, medical history and relevant family structure. This result should therefore be interpreted by appropriately qualified clinical personnel.

Consent should be obtained from the patient for release of these results.

Report Date and Time (GMT): 10/19/2022, 19:19

PGX Pipeline Version: 1.15.1

Operator Name: Test1
