# Supplementary material for: A New Cloud-Native Tool for Pharmacogenetic Analysis
Source: Genes (Basel). 2024 Mar 11;15(3):352. doi: 10.3390/genes15030352 (PMC10969787; doi:10.3390/genes15030352)
Supplement: Supplementary file 1 [file genes-15-00352-s001.zip › Pgxtools_ a new cloud-native tool for Pharmacogenetic analysis-Supplemental-1-revised.pdf]

Supplemental data

1. Variants in the database in Pgxtools

Supplemental Table S1. Genes and variants for PGx analysis

Gene	Drug(s)	Variants	
		cDNA	rs number
COMT	Opioid	c.472G>A	rs4680
CYP2B6	Efavirenz	c.983T>C	rs28399499
			rs34223104
		c.219-237A>G	rs776746
		c.432+2T>C	rs55965422
CYP2C19	Clopidogrel, Proton Pump Inhibitors (Omeprazole, Lansoprazole, Pantoprazole and Dexlansoprazole), Selective Serotonin Reuptake Inhibitors (Citalopram, Escitalopram and Sertraline), Tricyclic Antidepressants (Tertiary Amines Amitriptyline, Clomipramine, Doxepin, Imipramine and Trimipramine), Voriconazole	c.1A>G	rs28399504
		c.1297C>T	rs56337013
		c.395G>A	rs72552267
		c.819+2T>A	rs72558186
		c.358T>C	rs41291556
		c.431G>A	rs17884712
		c.680C>T	rs6413438
		c.-806C>T	rs12248560
		332-23A>G in the absence of 681G>A	rs12769205

Gene	Drug(s)	Variants	
		cDNA	rs number
		c.430C>T	rs1799853
		c.1075A>C	rs1057910
CYP2C9	Warfarin	c.1076T>C	rs56165452
		c.1080C>G	rs28371686
		c.818delA	rs9332131
		c.449G>A	rs7900194
		c.752A>G	rs2256871
		c.1003C>T	rs28371685
		c.1465C>T	rs9332239
		c.269T>C	rs72558187
		c.374G>A	
		c.485C>A	rs72558190
		c.895A>G	rs72558192
		c.1144C>T	
		c.1190A>C	rs72558193
		c.353_362del	rs1304490498
		c.389C>G	rs200965026
		c.641A>T	

Gene	Drug(s)	Variants	
		cDNA	rs number
		c.1429G>A	rs781583846
		c.395G>A	rs200183364
		374G>T + 430C>T	
		c.-1639G>A	rs9923231
		c.85G>T	rs104894539
CYP2D6	Ondansetron and Tropisetron, Selective Serotonin Reuptake Inhibitors (Paroxetine and Fluvoxamine), Opioid (Codeine, Tramadol and Hydrocodone), Atomoxetine, Tricyclic Antidepressants, Tamoxifen	c.775del	rs35742686
		c.506-1G>A	rs3892097
		c.454del	rs5030655
		c.971A>C	rs5030867
		c.505G>T	rs5030865
		c.886C>T	rs16947
		c.841_843del	rs5030656
		c.100C>T	rs1065852
		c.124G>A	rs5030862
		c.137dup	rs774671100
		c.320C>T	rs28371706
		c.805dup	rs72549352
		c.1012G>A	rs59421388

Gene	Drug(s)	Variants	
		cDNA	rs number
		c.406_408delins ATC	rs61736512+ rs1058164
		c.1319G>A	rs267608319
		c.320C>T	rs28371706
		c.985+39G>A	rs28371725
		c.1088_1089dup	rs72549346
		c.358T>A	rs1135822
		c.1030C>T	rs72549347
		c.975G>A	rs79292917
		c.785A>G	rs2279343
		c.516G>T	rs3745274
CYP3A5	Tacrolimus	c.624G>A	rs10264272
		c.1035dup	rs41303343
		c.82C>T	rs55817950
		c.1009G>A	rs28383479
CYP4F2	Warfarin	c.1457G>C	rs1135840
DPYD	Fluoropyrimidines 5-fluorouracil	c.557A>G	rs115232898
		c.1129-5923C>G	rs75017182
		c.1679T>G	rs55886062

Gene	Drug(s)	Variants	
		cDNA	rs number
		c.1905+1G>A	rs3918290
		c.2846A>T	rs67376798
		c.295_298delTCAT	rs72549309
		c.703C>T	rs1801266
		c.2657G>A	rs1801267
		c.2983G>T	rs1801268
		c.1003G>T	rs72549306
		c.238G>C	rs1800462
		c.460G>A	rs1800460
IL28B	PEG Interferon-Alpha-Based Regimens		rs12979860
NUDT15	Thiopurine	c.52G>A	rs186364861
		c.681G>A	rs4244285
		c.636G>A	rs4986893
SLCO1B1	Simvastatin	c.521T>C	rs4149056
		c.-910G>A	rs4149015
TPMT	Thiopurine (thioguanine, mercaptopurine and azathioprine)	c.719A>G	rs1142345
		c.626-1G>A	rs1800584

Gene	Drug(s)	Variants	
		cDNA	rs number
		c.146T>C	rs72552740
		c.644G>A	rs56161402
		c.374C>T	rs200220210
		c.415C>T	rs116855232
		c.416G>A	rs147390019
VKORC1	Warfarin	c.106G>T	rs61742245
		c.121G>T	
		c.134T>C	rs104894540
		c.172A>G	rs104894541
		c.196G>A	rs72547529
		c.358C>T	rs7200749
		c.383T>G	rs104894542
		c.1297G>A	rs2108622
		c.886C>T	rs16947

The table summarizes the genes, drugs and gene variants used in the Pgxtools. Gene variants are expressed in the form of cDNA change and rs number. Gene variants are selected from CPIC guidelines. Interpretations are made based on diplotypes in Pgxtools. The cDNA and rs numbers of SNPs are taken from allele definition tables in the CPIC guidelines. We have limited our scope to the single nucleotide polymorphisms (SNPs) in this article as the PGx significance of the SNPs are most studied and better understood. The other forms of variants such as large

Indels, CNVs would be considered when PGx guidelines and software algorithms for them are more mature.

2. Visualization of BAM/CRAM in G1K with EnsEMBL

To investigate why a small percentage of samples have different variants and / or genotypes reported by G1K and Pgxtools in Table 2, we performed a manual three-way comparison by visually inspecting alignments in a genome browser in EnsEMBL. The screenshot shows that there is a mixture of G and A at the position 42,130,692 in the diplotype of the Chromosome 22 in the NA19210.final.cram in G1K alignment (Supplemental figure 1). If G > 50%, we call it wild type and if A > 50%, we call it variant 22:42130692:G:A.

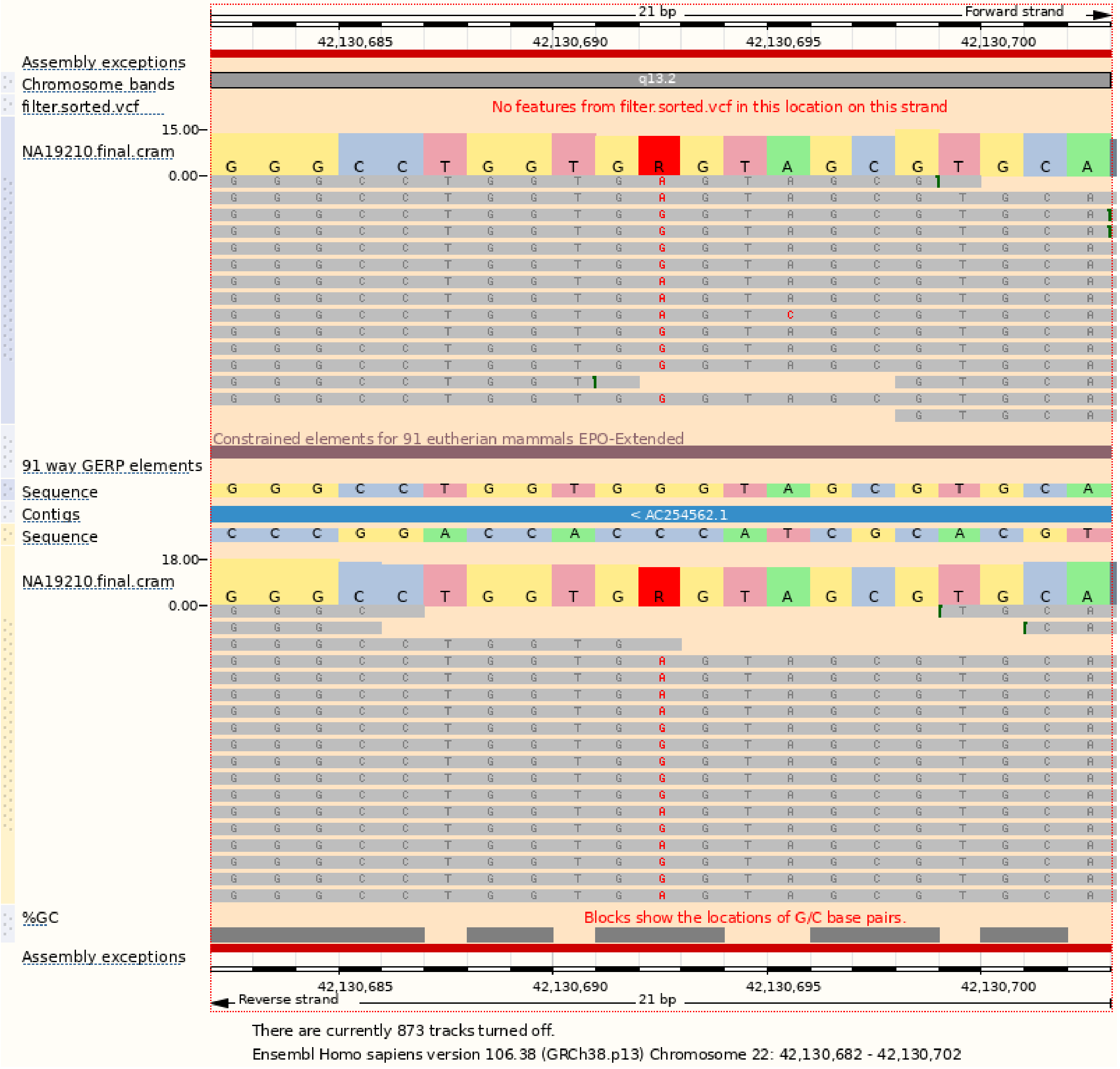

Supplemental Figure S1. G1K sample alignments NA19210.final.cram around 42130692

3. Graphic user interface

Screenshot of the graphic user interface of “[Supplemental Figure 2] JupyterLab graphic user interface.png”

Supplemental figure S2. Graphic user interface of Pgxtools.

4. PGx reports

(1) Single-gene-drug-pair report format

Sample report in a separate file of “[Supplemental Figure 3] HG01183_basic_report_TPMT_Thiopurine Azathioprine.html.pdf”.

Supplemental figure S3. Pharmacogenetics report on *TPMT* and thiopurine pair for sample HG01183.

(2) All-gene-summary report format

Sample report in a separate file of “[Supplemental Figure 4] HG01183_genes_summary_report.html.pdf”.

Supplemental figure S4. Pharmacogenetics summary report on all PGx gene variants found in sample HG01183.
