# Supplementary figures and images for: A New Cloud-Native Tool for Pharmacogenetic Analysis
Source: Genes (Basel). 2024 Mar 11;15(3):352. doi: 10.3390/genes15030352 (PMC10969787; doi:10.3390/genes15030352)

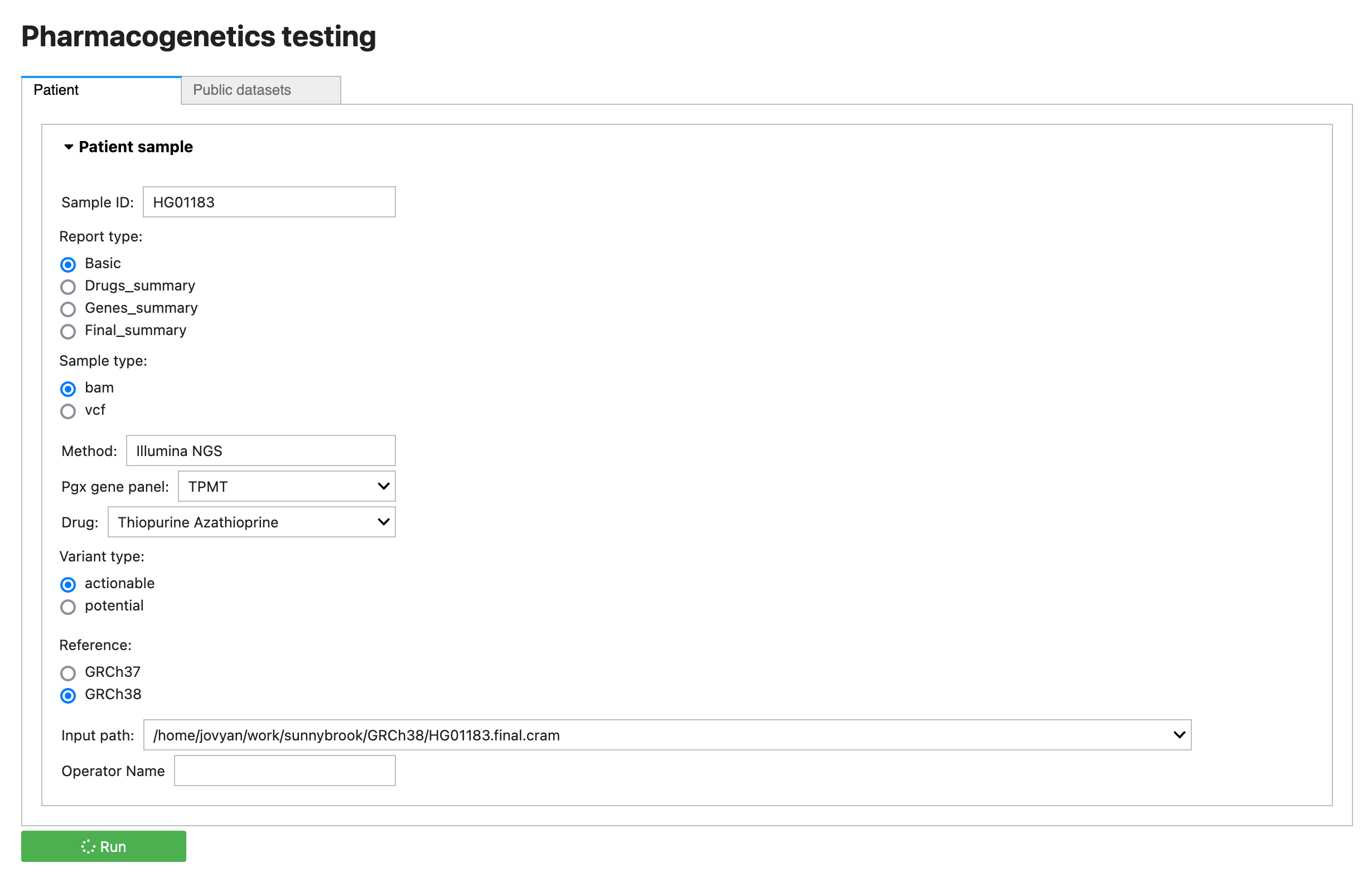

Supplement: Supplementary file 1 [file genes-15-00352-s001.zip › [Supplemental Figure S2] JupyterLab graphic user interface.png]
